# Ultrasmall SnS_2_ quantum dot−based photodetectors with high responsivity and detectivity

**DOI:** 10.1515/nanoph-2022-0277

**Published:** 2022-09-29

**Authors:** Yi Ren, Hua An, Weiguan Zhang, Songrui Wei, Chenyang Xing, Zhengchun Peng

**Affiliations:** Key Laboratory of Optoelectronic Devices and Systems of Ministry of Education and Guangdong Province, School of Physics and Optoelectronic Engineering, Shenzhen University, Shenzhen 518060, P. R. China; School of Mechatronics and Control Engineering, Shenzhen University, Shenzhen 518060, P. R. China; Interdisciplinary Center of High Magnetic Field Physics of Shenzhen University, School of Physics and Optoelectronic Engineering, Shenzhen University, Shenzhen 518060, P. R. China

**Keywords:** PEC-type photodetectors, quantum dots, tin disulfide

## Abstract

Quantum dots (QDs) often exhibit unique behaviors because the reduction in lateral size leads to stronger quantum confinement effects and a higher surface-to-volume ratio in comparison with larger two-dimensional nanosheets. However, the preparation of homogeneous QDs remains a longstanding challenge. This work reports the preparation of high-yield and ultrasmall tin disulfide (SnS_2_) QDs by combining top–down and bottom–up approaches. The as-prepared SnS_2_ QDs have a uniform lateral size of 3.17 ± 0.62 nm and a thicknesses 2.39 ± 0.88 nm. A series of self-powered photoelectrochemical-type photodetectors (PDs) utilizing the SnS_2_ QDs as photoelectrodes are also constructed. Taking advantage of the tunable bandgaps and high carrier mobility of the SnS_2_, our PDs achieve a high photocurrent density of 16.38 μA cm^−2^ and a photoresponsivity of 0.86 mA W^−1^, and good long-term cycling stability. More importantly, the device can display obvious photoresponse, even at zero bias voltage (max), and greater weak-light sensitivity than previously reported SnS_2_-based PDs. Density functional theory calculation and optical absorption were employed to reveal the working mechanism of the SnS_2_ QDs-based PDs. This study highlights the prospective applications of ultrasmall SnS_2_ QDs and provides a new route towards future design of QDs-based optoelectronic devices.

## 1 Introduction

Recently, photodetectors (PDs) based on two-dimensional (2D) materials such as graphene [1, 2], black phosphorus (BP) [3, 4], and layered metal dichalcogenides (LMDs) [5, 6] have been active areas of research because of their unique optoelectronic properties. Among these PDs, graphene is the most studied owing to the high carrier mobility (≈10^5^ cm^2^ V^−1^ s^−1^) [7]. However, graphene-based photoelectric devices are greatly limited by their absence of a bandgap. Different from graphene, BP shows great promise for optoelectronic applications benefits from its widely tunable energy gap (from 0.3 eV in the bulk to 2.2 eV for a monolayer) [8]. Nevertheless, BP-based devices are susceptible to degradation under deleterious environmental conditions because of its oxygenated and hydrophilic properties [9]. LMDs encompass a large family of materials (e.g. ReS_2_, MoS_2_, NbSe_2_, CrSe_2_, SnS_2_, and WS_2_), and many of them exhibit landmark features that subjected to an indirect–direct bandgap transition from the bulk phase to a monolayer [10–12]. In addition, the high mobility and unique size, thickness, and flexibility of LMDs result in excellent electronic properties, such as the improved integration levels and containment of short-channel effects [13–15]. These merits render LMDs suitable candidates for high-next-generation optoelectronics. Among various semiconducting LMDs, SnS_2_ is a promising material for electronic and optoelectronic applications with high charge carrier mobility, regulable bandgap (from 2.41 eV for a monolayer nanosheet to 2.18 eV in the bulk phase), and a high absorption coefficient of 10^4^ cm^−1^ [16]. However, the performance of SnS_2_−based PDs in terms of responsivities and detectivities remains poor, which hinders their practical applications [17–19].

Researchers have been made extensive efforts to improve the performance of LMDs-based PDs by constructing heterostructures [20, 21], morphology engineering [22–24] and chemical doping [25, 26]. In particular, tailoring the lateral dimensions of LMDs to quantum dots (QDs) or 0D nanodots is an effective means of adjusting their intrinsic properties or inducing new optical/electrical characteristics [27]. Among these LMDs-based nanostructures, QDs possess many inherent advantages in the field of PDs such as strong quantum confinement effects, large surface area to volume ratios, and additional active edge sites [28, 29]. Compared with widely studied MoS_2_ and WS_2_ [30–32], reports on mild preparation methods for SnS_2_ QDs remain scarce and incomprehensive. So far, SnS_2_ QDs have been synthesized using two classes of methods: top-down method by high-powered and long-time liquid exfoliation [33] and bottom–up route by L-cysteine-assisted hydrothermal method [34]. Nevertheless, the low yield and repeatability, complicated syntheses procedures and lack of redispersed solvents remain bottlenecks, leading to uncontrollable and irreproducible reactions from the various batches. Therefore, a strategy for mass production and reproducible preparations of high-quality of SnS_2_ QDs is highly desirable.

Herein, we overcome this performance bottleneck by combining both top–down and bottom–up approaches through liquid-phase exfoliated (LPE) and solvothermal methods for the fabrication of ultrasmall and high-quality SnS_2_ QDs with outstanding self-powered PD properties. At zero bias condition, the devices exhibited a high photocurrent density (*P*_ph_) of 0.72 μA cm^−2^ in 0.1 M KOH. Furthermore, the photo-response behavior of photoelectrochemical (PEC) SnS_2_ QDs-based PDs is studied under different electrolyte, light wavelength, and bias potential and its possible working mechanism as revealed by Density functional theory (DFT) calculations for the first time. In addition, the SnS_2_ QDs-based PDs also exhibit good long-term stability over 30 days, which was unimaginable for analogous QD materials. Our work provides a facile method to obtain high quality of SnS_2_ QDs, which provides us with new perspectives for designing novel QDs-based PEC PDs.

## 2 Experimental section

### 2.1 Synthesis of 2D and 0D SnS_2_ nanostructures

Briefly, SnS_2_ NSs were prepared from SnCl_4_·5H_2_O and thioacetamide (TAA) by a hydrothermal method in 20 mL of DI water. The autoclave was sealed and maintained at 160 °C for 12 h in an electric oven. After the hydrothermal reaction, the obtained SnS_2_ NSs were washed with water and ethanol several times. Subsequently, SnS_2_ powder was sonicated in NMP solvent at a concentration of 6 mg mL^−1^ for 8 h. The few-layer SnS_2_ was obtained and centrifuged at a speed of 6000 rpm for 5 min. Thereafter, the obtained suspension was delivered to a three-neck flask under argon and heated in an oil bath at 140 °C for 3 h. Thus, high−quality SnS_2_ QDs with uniform size were formed. Before the devices were fabricated, the SnS_2_ QDs were stored in the NMP solution.

### 2.2 Characterization

The morphologies of the SnS_2_ QDs were investigated by TEM and high-resolution TEM (JEOL-JEM-2100F) and AFM (Shimadzu SPM-9700, Dimension instrument with 512−px resolution). Powder XRD was carried out by a PhilipsX’Pert Pro Super diffractometer with Cu–Ka radiation (*λ* = 0.154 nm) at scanning rate of 5° min^−1^. Raman spectra were examined on a high-resolution confocal Raman spectroscopy (HR Evolution) with a 633 nm laser excitation. The S 2*p* and Sn 3*d* envelope were characterized by XPS (Thermo K-alpha+). UV–vis absorption spectra were collected on a shimadzu-3600plus spectrometer.

### 2.3 Device fabrication

First, ITO substrates were washed ultrasonically with acetone, ethanol, and DI water in order for 20 min total. After drying in nitrogen (N_2_), 1 mg of SnS_2_ QDs was added to 1 mL of Nafion/NMP (0.05 wt%) and sonicated for 30 min (320 W) to obtain a homogeneous slurry. Thereafter, the mixture was dropped onto the conductive side of ITO glass, which was dried in a vacuum oven at 80 °C overnight.

### 2.4 Photoresponse measurement

The photo-response behavior of SnS_2_ QDs was evaluated with a photoelectrochemical (PEC) system on an electrochemical workstation (CHI 760E). The SnS_2_ QDs-coated ITO glass, platinum plate, and a KCl saturated Ag/AgCl as the working electrode, counter electrode, and reference electrode, respectively. The SnS_2_ QDs-based devices were illuminated under different simulated lights (mixed light from 300 to 800 nm; monochromatic light: 365, 380, 400, 475, and 550 nm) and applied in five electrolytes (0.1, 0.5, 1.0 M of KOH; 0.1 M Na_2_SO_4_; and 0.1 M HCl). The linear sweep voltammetry measurements were collected from 0.0 to 1.0 V at a scanning rate of 10 mV/s. The electrochemical impedance spectroscopy was performed over a frequency range from 1 to 100 KHz with amplitude of 0.005 V in the dark. The photocurrent−time (I−T) curves were collected at bias voltages from 0 to 0.6 V with an interval of 5 s.

### 2.5 DFT calculations

DFT calculations were conducted by using the Vienna Ab-inito Simulation Package (VASP) [35]. The spin polarized Perdew−Burke−Ernzerhof (PBE) exchange-correlation functional and projector augmented wave method was used [36, 37]. The cutoff energy of 500 eV and the Monkhorst−Pack *k*-point mesh of 11 × 11 × 7 for the primitive unit cell of bulk structure and 11 × 11 × 1 for slab model (1, 2, 4, 8 and 12 layers) were used to ensure that atomic positions and lattice constants were fully relaxed until total energy difference and forces were less than 10^−4^ eV and 0.01 eV/Å, respectively [38]. The D3 correction with Becke–Johnson damping was included to correct van der Waals interaction in multi-layers structures, close to the experimental value of SnS_2_ [39, 40]. A higher convergence criterion for total energy, 10^−6^ eV, was adopted to calculate electronic structures. The periodic boundary condition was applied in all three directions. A vacuum space of at least 15 Å was applied in the *z*−direction to avoid the interaction between two periodic images in each slab model.

## 3 Results and discussion

### 3.1 Morphology and crystal characterization of SnS_2_ QDs

**Scheme 1:**
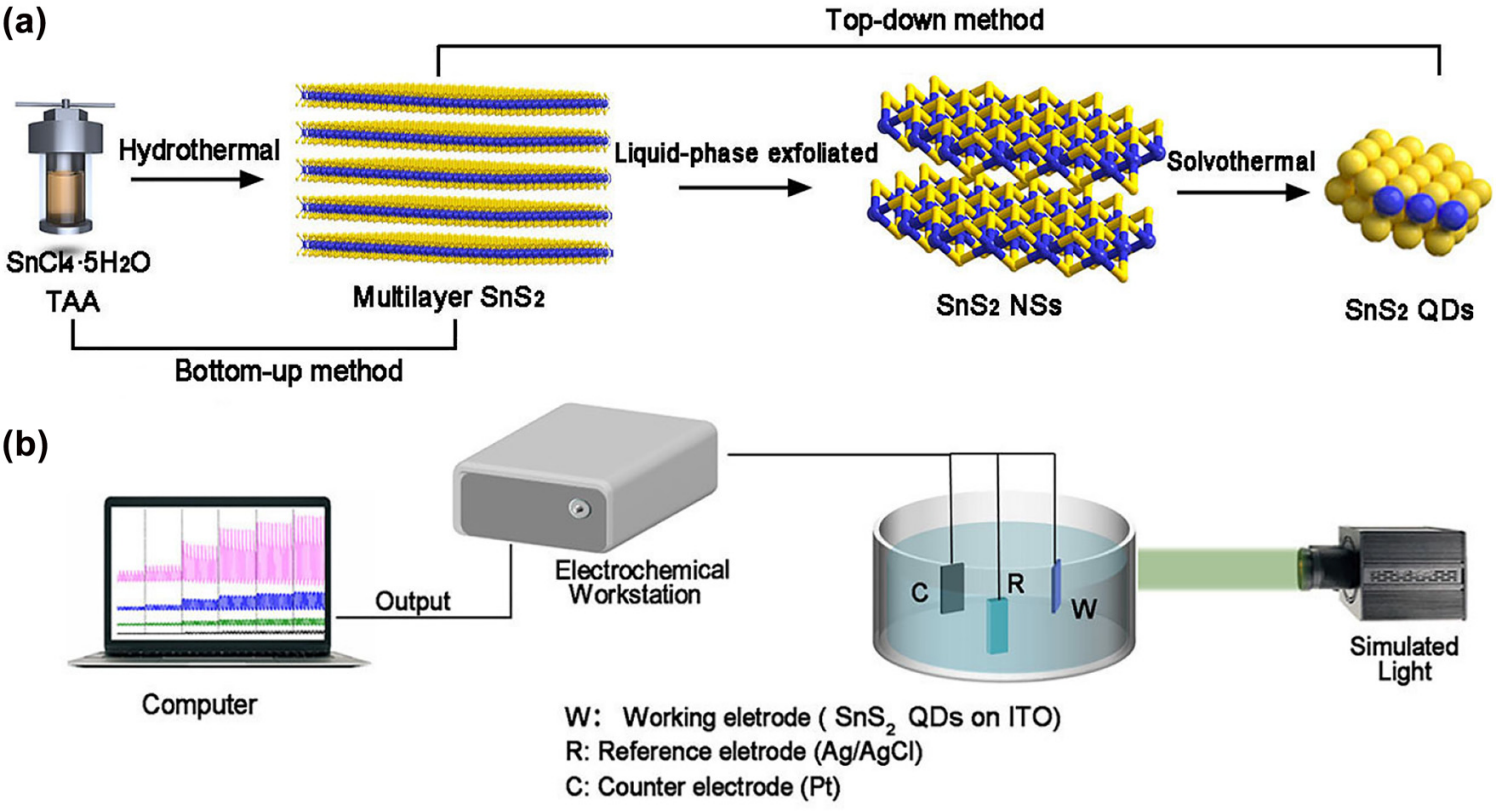
Preparation process of the SnS_2_ QDs and SnS_2_ QDs-based PDs. (a) Preparation process of the SnS_2_ QDs. (b) Schematic Diagram of evaluating SnS_2_ QDs PDs.

**Figure 1:**
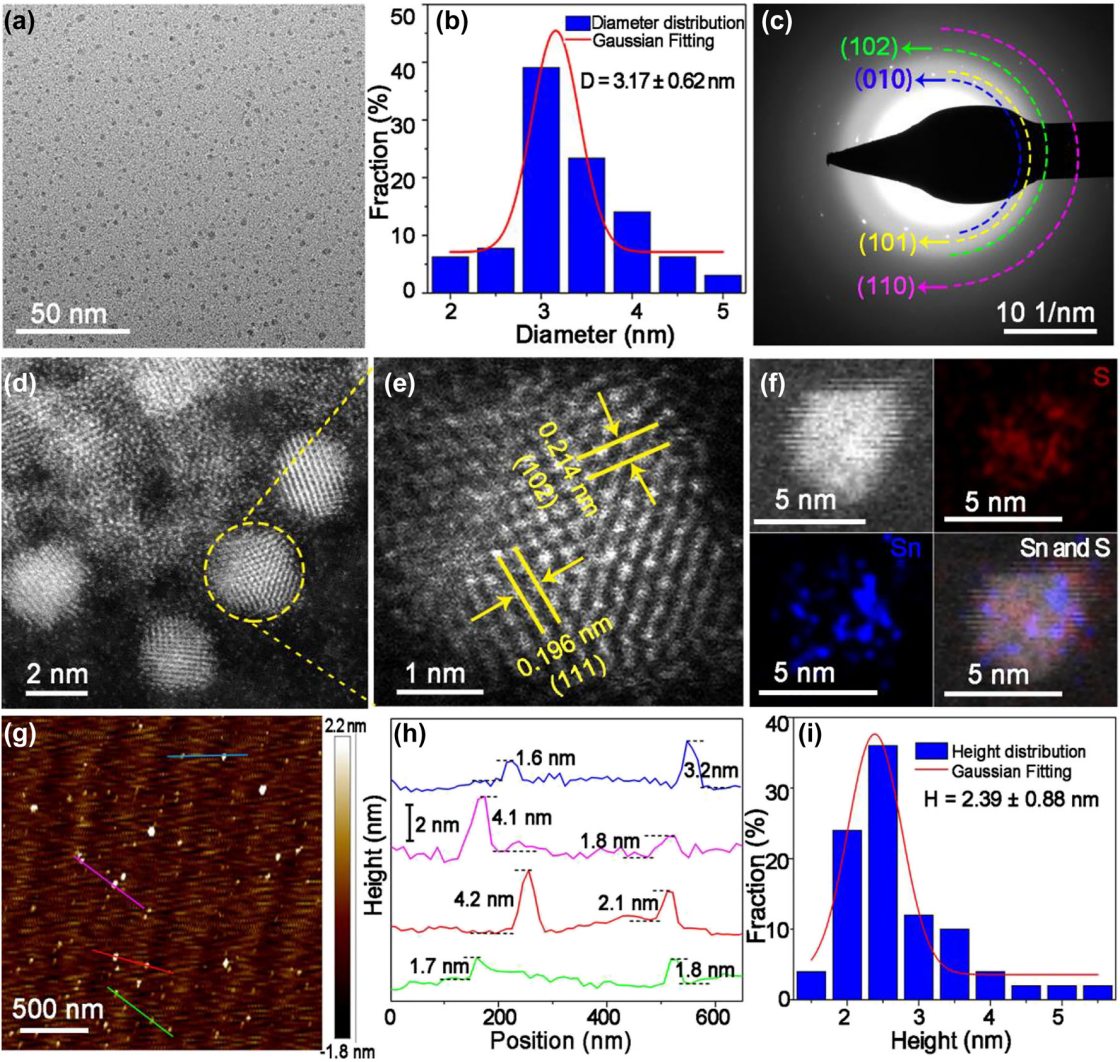
Morphology and characterizations of SnS_2_ QDs. (a) TEM images of as-synthesized SnS_2_ QDs. (b) Lateral size distribution of SnS_2_ QDs. (c) Typical SAED image of SnS_2_ QDs. (d, e) HAADF-STEM image of SnS_2_ QDs. (f) Corresponding EDS mapping of SnS_2_ QDs. (g) AFM image of a resultant SnS_2_ single crystal. (h) Thickness distribution of SnS_2_ QDs. (i) Vertical size distribution of SnS_2_ QDs.

Scheme 1 demonstrates a representative procedure for SnS_2_ QDs-based PEC-type PDs. SnS_2_ nanosheets (NSs) were obtained through LPE of multilayer SnS_2_ by using the strongly-polar solvent N-methyl-2-pyrrolidone (NMP) for dispersion. Then, uniform-sized SnS_2_ QDs were successfully fabricated by increasing solvothermal method, and this approach could effectively improve the quality of SnS_2_ QDs. Subsequently, SnS_2_ QDs was deposited on the indium tin oxide a (ITO) glass by drop−coating and used the SnS_2_ QDs as the working electrode of a standard triple electrode for PEC-type PDs (refer to the experimental section for details). The transmission electron microscopy (TEM) image in Figure 1a indicates quite uniform SnS_2_ QDs with a circular shape. It shows the distribution of SnS_2_ QDs particles with a diameter at ca. 3.17 nm (Figure 1b). The typical high-angle annular dark-field scanning transmission electron microscopy (HAADF-STEM) image of SnS_2_ QDs is depicted in Figure 1d. The crystal lattices of SnS_2_ QDs were 0.196 and 0.214 nm (Figure 1e), corresponding to the (111) and (102) faces of hexagonal SnS_2_ crystals (JCPDS card No. 23-677) [41]. The results of the corresponding selected area electron diffraction (SAED) pattern (Figure 1c) indexed well to the hexagonal SnS_2_ crystal and the marked lattice planes are in good agreement with the previous report [42, 43]. Energy-dispersive X-ray spectroscopy (EDS) elemental mapping (Figure 1f) further illustrates the successful synthesis of SnS_2_ QDs. Besides, Figure S1 indicates that individual SnS_2_ NSs were hexagonal structures with lateral dimensions about 20 nm. The morphology and thickness of the as-prepared SnS_2_ QDs was further investigated by atomic force microscopy (AFM) image in Figure 1g. The average thickness of the SnS_2_ QDs ranged from 0.62 nm (one layer) to 6.24 nm (10 layers), with an average thickness of 2.39 nm, indicating their predominantly four-layer nature (since the thickness of a monolayer was 0.60 nm) [44, 45].

In addition, the suspension state of the as-prepared SnS_2_ QDs was very stable, with no significant aggregation and decomposition for at least 1 month, probably due to the protective chemical stability of the NMP solvent for SnS_2_, similar to that of BP (Figure S2) [46].

**Figure 2:**
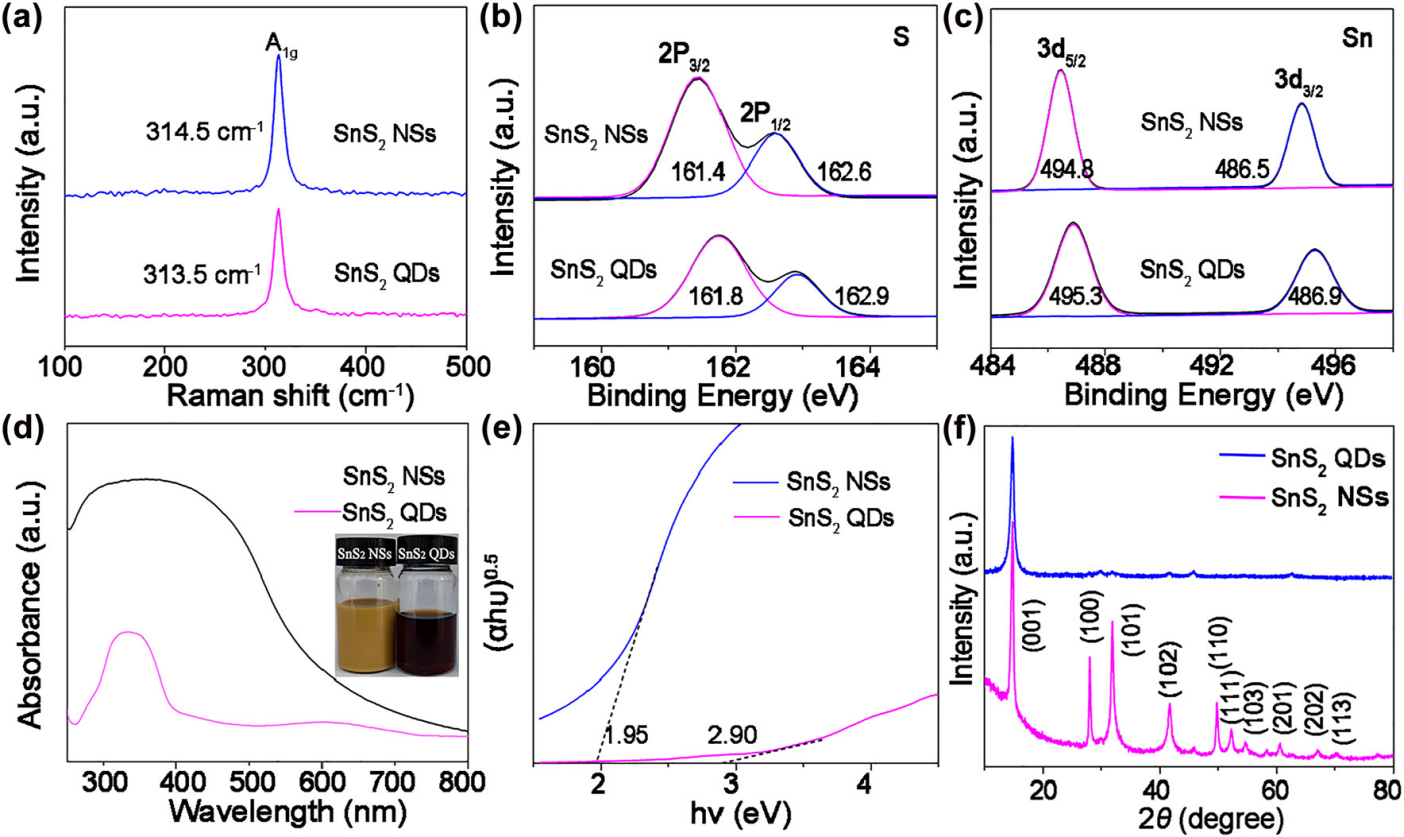
The characterization of SnS_2_ QDs. (a) Raman spectra of 2D and 0D SnS_2_ at 532 nm excitation wavelength. (b, c) XPS spectra of S 2p and Sn 3d of SnS_2_ QDs and NSs, respectively. (d) UV–visible absorption spectra. The inset shows the photographs of as-prepared SnS_2_ NSs and SnS_2_ QDs solutions. (f) XRD patterns of the SnS_2_ QDs and SnS_2_ NSs.

In the Raman spectra of 2D and 0D SnS_2_ as shown in Figure 2a, the strong characteristic peaks at 314.5 and 313.5 cm^−1^ correspond to the A_1g_ vibration mode of SnS_2_ NSs and SnS_2_ QDs, respectively. This observed in−plane Raman mode of the atomic vibration shift at ca. 1 cm^−1^ closely correlates with the substantial variation of nanostructured SnS_2_, which is consistent with the literature [41, 47]. Furthermore, the elemental composition and binding information of SnS_2_ were intensively studied by high-resolution X-ray photoelectron spectroscopy (XPS). Figure 2b and c present the comparison of high-resolution XPS spectra of S 2*p* and Sn 3*d* of the as-prepared SnS_2_ NSs and SnS_2_ QDs samples. Regarding the S 2*p* spectra (Figure 2b), the peaks located at 161.8 and 162.9 eV are indexed to S 2*p*_3/2_ and 2*p*_1/2_ orbitals of SnS_2_ QDs. The peak position of S 2*p* shows gradually positive shift with the decrease in the lateral size of SnS_2_. The peaks at 495.3 and 486.9 eV are attributed to the Sn 3*d*_3/2_ and Sn 3*d*_5/2_ orbitals of SnS_2_ QDs. The similar increase of the binding energy observed in the Sn 3*d* X-ray photoelectron spectra (Figure 2c).

The morphology-dependent properties of SnS_2_ have been investigated by UV−vis. Figure 2d shows the UV–vis spectra of SnS_2_ NSs and QDs in NMP. It can also be clearly observed that SnS_2_ NSs show absorption ranging from 250 to 800 nm (Figure 2d) and the corresponding bandgap was estimated to be ca. 2.90 eV when taking (Ahν)^0.5^ as a function of the photon energy (hν; Figure 2e), which is in agreement with a previous report [48]. After the reaction, the solution changed from brown yellow to a transparent purple, and the final product was collected by centrifugation. The main peak of SnS_2_ QDs was blue-shifted compared to SnS_2_ NSs. These results originated from the quantum confinement of carriers, similar results were reported in MoS_2_ and WS_2_ QDs [49]. The crystal structure of the obtained SnS_2_ NSs and SnS_2_ QDs were systematically investigated by X-ray powder diffraction (XRD) patterns. As shown in Figure 2f, the peak position of SnS_2_ QDs are almost the same compared to the XRD pattern of SnS_2_ NSs, although the characterized (001) reflection was broadened and most of other diffraction intensities were obviously reduced, indicating the highly exfoliated nature of these QDs [50].

### 3.2 DFT calculations of SnS_2_

**Figure 3:**
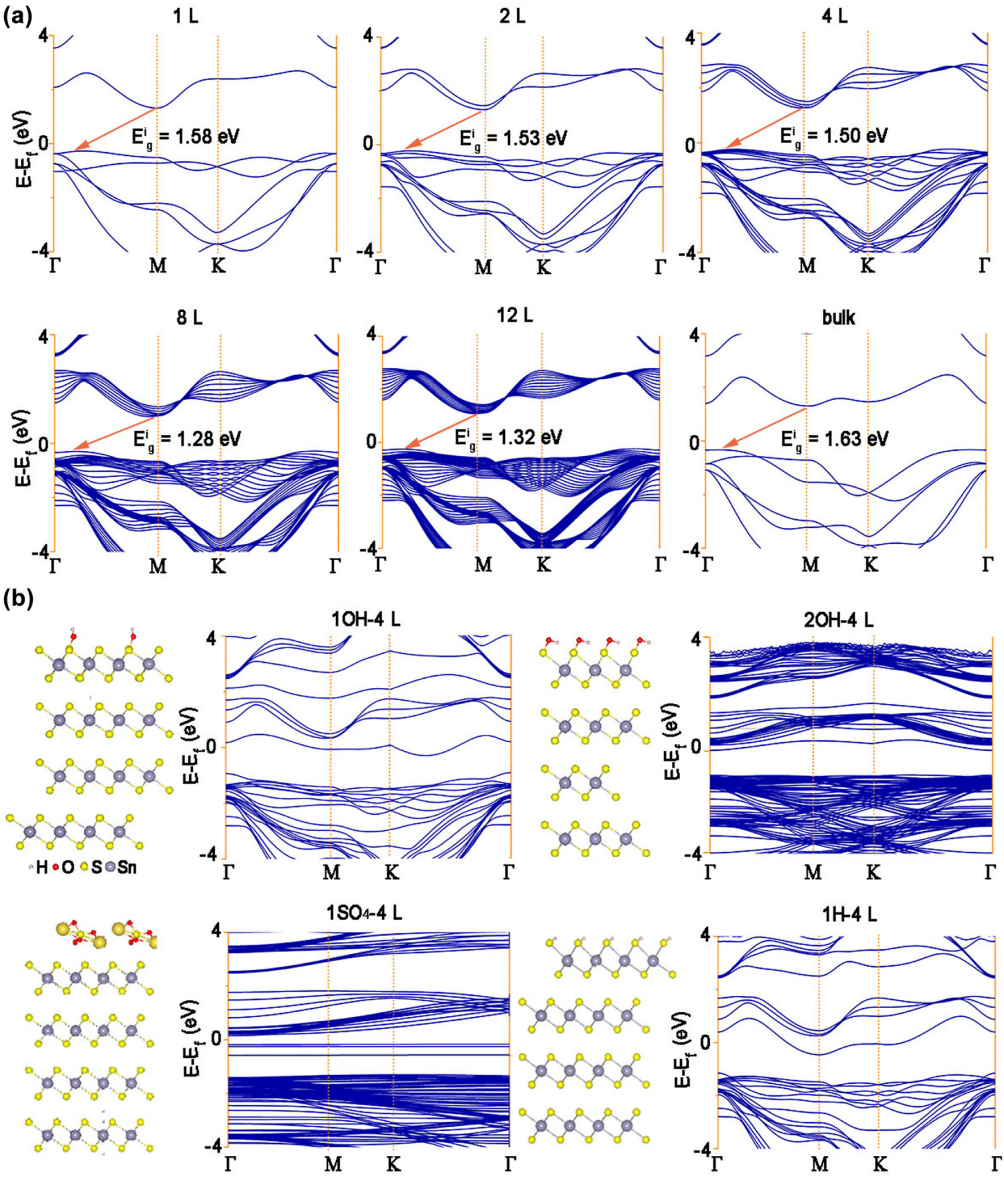
Calculated band structures of SnS_2_ with different layers. (a) Calculated band structures of SnS_2_ with layer number of 1, 2, 4, 8 and 12 layers. (b) Calculated band structures of 4L SnS_2_ coordinated with 1OH, 2OH, 1SO_4_, 1H groups.

Although monolayers of SnS_2_ has been considered by several theoretical [16, 44] and experimental groups [39, 51, 52], the SnS_2_ QDs with few-layer have not been systematically studied so far. To gain further insight, this work investigates the evolution of the structural and electronic properties of SnS_2_ based on DFT calculations, by using Heyd–Scuseria–Ernzerhof (HSE06) hybrid functional and Perdew−Burke−Ernzerhof (PBE) methods. In contrast to MoS_2_, both bulk SnS_2_ and few-layer SnS_2_ are indirect band gap semiconductors [53, 54]. As indicated by the layer dependence of the band structure of SnS_2_ (Figure 3), the conduction-band minimum (CBM) was located at the M high-symmetry point, and the valence-band maxima (VBM) was at a point along the Γ−M line. Our calculations indicate the thickness-dependence of theoretical indirect band gap (e.g.) of SnS_2_ which ranged from 1.32 eV for 12 layers to 1.588 eV for the monolayer, see details in Table S1. As displayed in Figure 3a, the decrease of e.g. with increasing number of layers, which can be credited to the effective reduction of electrostatic interactions by screening the vacuum in the lamellar structure, as well as the quantum confinement of electrons within a quasi-2D material of finite thickness [55]. Notably, using PBE methods alone to describe exchange-correlation interaction of electrons will undervalues the e.g. of the material (≈1 eV), since it ignores the screened Coulomb potential for Hartree–Fock exchange [56, 57]. Hence, e.g. of one-layer, four-layer and bulk SnS_2_ were also calculated by using hybrid HSE06 functionals (Figure S3), and obtained values of 2.42, 2.30 and 2.48 eV, respectively, similar to the values of e.g. calculated from the UV–vis spectra (see Figure 2e). As illustrated in Figure S3d, the total and local density of states (DOS) of four−layer SnS_2_ indicates that the VBM were dominated by S-3*p* states, and a large DOS of the VBM was considered as the main contribution to the high photo-conversion efficiency. In contrast, the S-3*p* and Sn-5*s* hybridized states played dominant roles in the CBM.

The e.g. has an important influence on the photo-response behavior of SnS_2_ QDs-based PDs, where a decreased e.g. can enhance the absorption of light energy and result in better photo-response performance. To further elaborate on the electrolyte concentration and different electrolytes effects, the band gaps of SnS_2_ are calculated under different concentrations and electrolytes, respectively. On the basis of the aforementioned AFM results, the thickness of the prepared SnS_2_ QDs was mainly four layers. Therefore, four−layers of SnS_2_ QDs was selected as the thickness for further study. As displayed in Figure 3b, the Eg of SnS_2_ QDs were substantially modulated with different external electrolyte. For instance, the Eg of low concentration of hydroxyl terminations (1−OH) adsorbed onto four−layer SnS_2_ QDs was evaluated to be 0.55 eV, which is remarkably lower than that of pristine four−layer SnS_2_ QDs (1.50 eV). Therefore, the photoresponse performance of PDs can be significantly improved by increasing the KOH concentration, which is in good accordance with the experimental results.

### 3.3 Optoelectronic performance of SnS_2_ QDs

**Figure 4:**
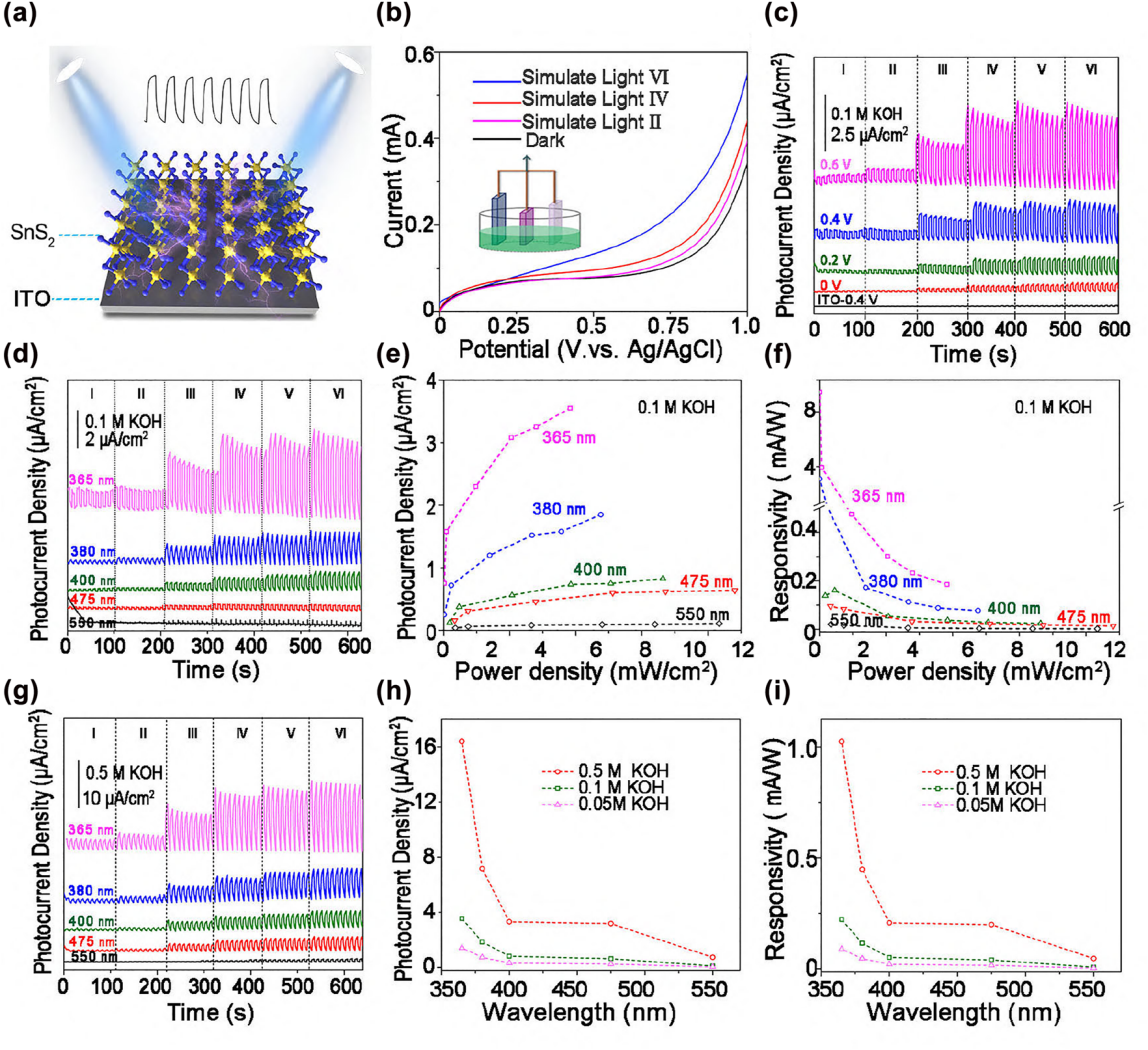
The photoresponse behavior of SnS^2^ QDs under different conditions. (a) Structural diagram of working electrode (SnS_2_ QDs onto the ITO glass). (b) LSV curve of SnS_2_ QDs-based PDs in different level of simulate light. (c) Photo-response behavior under mixed light in 0.1M KOH at different bias potentials. (d) Typical on/off switching behavior in 0.1M KOH under different monochromatic light at 0.6 V. The calculated (e) photocurrent density (*P*_ph_) and responsivity (*R*_ph_) as a function of power density at 0.6 V (g) photo-response behavior in 0.5 M KOH under different monochromatic light at 0.6 V. The calculated (h) *P*_ph_ and (i) *R*_ph_ under various wavelengths as the increment of KOH concentration at level *VI*.

Based on the theoretical investigations of the layer-dependent electronic structure of SnS_2_ and UV−vis absorption spectra, four−layer SnS_2_ QDs in KOH electrolyte with a small bandgap by UV can be easily excited by UV light to generate electrons and enhance their electrochemical properties. Consequently, SnS_2_ QDs were utilized as candidate material for PEC-type PDs. Figure 4a shows a schematic diagram of the SnS_2_ on the ITO and employed as the working electrode of the (PEC-type) PDs. Generally, the pertinent parameters of photoresponse characteristics including photocurrent density (*I*_ph_), photoresponsivity (*R*_ph_), and specific detectivity (D*) by the following equations [58].

(1) $$I_{\text{ph}}=\left(I_{\text{light}}-I_{\text{dark}}\right)/S$$

(2) $$R_{\text{ph}}=I_{\text{ph}}/P_{\lambda }$$

(3) $$D^{*}=R_{\text{ph}}\times S^{1/2}/{\left(2q\times I_{\text{dark}}\right)}^{1/2}$$

where *I*_light_ and *I*_dark_ denote the current with and without light, respectively; *P*_λ_ is the light power density; *S* is the effective area of SnS_2_ sample on ITO glass (2 cm^2^) under illumination; and *q* is the electron charge constant with a value of 1.60 × 10^−19^ C.

The linear sweep voltammetry (LSV) measurements of SnS_2_ QDs were conducted in the range of 0–1.0 V under conditions of darkness and simulated light irradiation with different intensities (Figure 4b). There was no obvious redox peak under a bias voltage of 0–0.6 V, which indicates SnS_2_ remain stable within bias voltage range. The photoresponse characteristics of SnS_2_ QDs-based phtodetectors were systematically studied at increased light power density (*P*_λ_) and different electrolyte solutions. Figure 4c the photocurrent density (*P*_ph_) as a function of both the bias voltages and light power density (*i.e.*, from level I to level VI, see details in Table S2), under simulated light (a mixed light from 300 to 800 nm), *P*_ph_ increased from 0.72 to 1.5, 2.94, and 7.1 μAcm^−2^ at Level VI, and the applied bias potential was increased from 0 to 0.2, 0.4 and 0.6 V, respectively. The *P*_ph_ values were substantially enhanced with ∼10 times, because a higher bias will promote photogenerated carrier efficiency. Notably, a stable “on–off” switching behavior of current density is clearly observed even at unbiased potentials, confirming its promising applications in the self-powered PDs. In addition, the on/off signal of uncoated ITO glass in 0.1 M KOH was negligible, which demonstrates the photoresponse signal under simulated light originated from the SnS_2_ QDs.

On the basis of the UV−vis near-infrared absorption spectra of SnS_2_ QDs, the absorption peak was ranged from 250 to 550 nm. Therefore, five quasi-monochromatic light wavelengths (*λ* = 365, 380, 400, 475 and 550 nm) were used for evaluating the photoresponse performance of SnS_2_ QDs-based PDs. Figure 3d and e display the *P*_ph_ the relationships with *P*_λ_ under the light irradiation at various wavelengths and a bias potential of 0.6 V in 0.1 M KOH. It can be observed that *P*_ph_ shows a decreasing trend from 365 to 550 nm. At level *VI*, *P*_ph_ reached 3.55 μAcm^−2^ (365 nm) and decreased to 0.11 μAcm^−2^ (550 nm). This result is in good accordance with the UV–vis spectra, and the same phenomena can be observed in 0.5 M KOH electrolyte (Figure 4g). In contrast to the relationship of *P*_ph_ − *P*_λ_, *R*_ph_ exhibited an opposite trend as shown in Figure 4f. Under the irradiation of 365 nm laser, *R*_ph_ rapidly decreased from 10.27 mA W^−1^ (level *I*, 0.073 mW cm^−2^) to 3.95 mA W^−1^ (level *II*, 0.40 mW cm^−2^), 0.47 mA W^−1^ (level *III*, 4.85 mW cm^−2^), 0.30 mA W^−1^ (level *IV*, 10.25 mW cm^−2^), 0.23 mA W^−1^ (level *V*, 14 mW cm^−2^), and further to 0.19 mA W^−1^ (level *VI*, 19.15 mW cm^−2^). As illustrated in Figure 4h, the relationship between *P*_ph_ and electrolyte concentration was also evident, with an 2.5 and 16.7 − fold increase in *P*_ph_ when the KOH concentration was increased from 0.05 M to 0.1 and 0.5 M at level VI, respectively. In addition, the calculated D* value was 7.02 × 10^8^ Jones under an illumination of 365 nm (level *VI*) in 0.1 M KOH at 0.6 V, lower than that in a high concentration of KOH electrolyte (5.83 × 10^9^ Jones in 0.5 M KOH). These results can be explained by the fact that both an increased bias potential and KOH concentration can improve the photoresponse due to the promotion of electron excitation and the increased ion concentration, respectively. The maximum *P*_ph_ value of 16.38 μA cm^−2^ is obtained under the irradiation of 350 nm monochromatic light in 0.5 M KOH, which is significantly higher than most of previously reported PEC-type PDs, as summarized in Table 1. The results indicating the excellent PEC photoresponse behavior of the 2D SnS_2_ QDs-based PDs in the this work.

**Table 1:** The comparison of the SnS_2_ QDs-based PDs and other previously reported PEC-type photodetection.

Materials	Conditions	Photocurrent (µA/cm^2^)	Responsivity (mA/W)	Detectivity (*D* ^*^, Jones)	Ref.
α-Ga_2_O_3_	0.5 M Na_2_SO_4_, 0 V	19.49	1.44	–	[43]
PbO QDs	0.01 M KOH, 0.6 V	5.93	4.28	–	[59]
PbS QDs	0.1 M KOH, 0.4 V	12.89	10.97	3.96 × 10^10^	[60]
Bi_2_S_3_ NSs	0.1 M KOH, 0.6 V	11.00	0.052	3.75 × 10^8^	[61]
SnS NSs	0.1 M Na_2_SO_4_, 0.6 V	1.59	0.060	1.92 × 10^8^	[62]
GeS NSs	0.5 M KOH, 0.6 V	8.57	0.032	3.71 × 10^7^	[63]
Bi_2_O_2_S	1.0 M KOH, 0.6 V	9.56	13.00	2.34 × 10^10^	[64]
InSe NSs	0.2 M KOH, 1.0 V	0.38	0.005	–	[65]
GeSe NSs	0.1 M KOH, 0.3 V	4.40	43.60	6.28 × 10^10^	[66]
PbSe NCs	0.1 M KOH, 0.4 V	11.88	12.37	–	[67]
Bi_2_Te_3_ NPs	0.5 M KOH, 0.6 V	8.68	0.40	–	[68]
SnS_2_ QDs	0.1 M KOH, 0.6 V	3.55	0.19	7.02 × 10^8^	This work
SnS_2_ QDs	0.5 M KOH, 0.6 V	16.38	0.86	5.83 × 10^9^	This work

**Figure 5:**
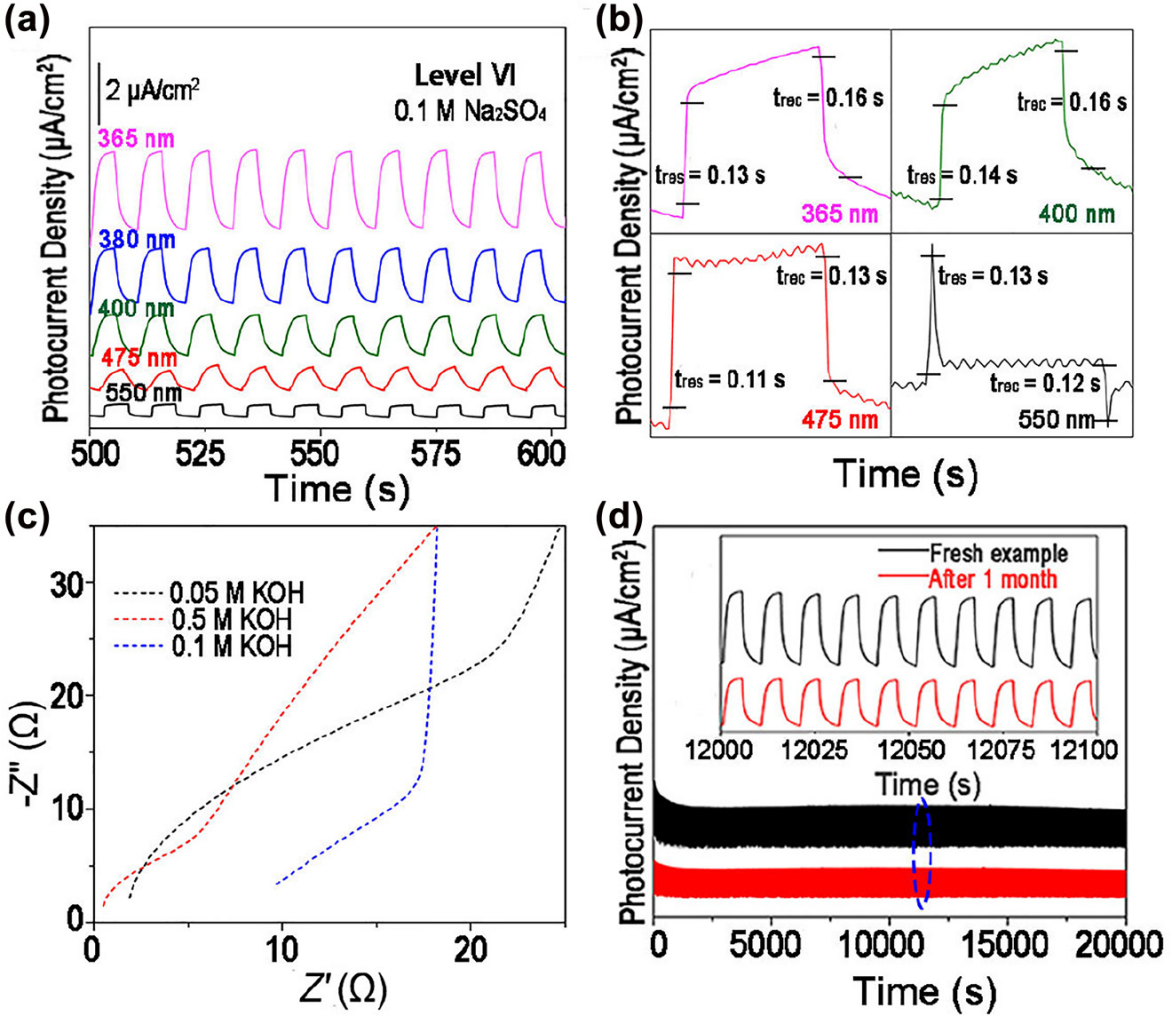
Photoresponse characteristics of SnS_2_ QDs-based photodetectors. (a) Photoresponse characteristics of SnS_2_ QDs-based PDs in 0.1M Na_2_SO_4_ electrolyte at 0.6 V bias and level *VI*. (b) Under the irradiation of varying wavelengths, the *t*_res_ and *t*_rec_ of SnS_2_ QDs-based PDs were abtained in 0.1 M KOH at level *VI* and 0.6 V. (c) EIS results of SnS_2_ QDs-based PDs in KOH electrolytes with different concentrations under the darkness. (d) Stability of on/off switching behaviors of SnS_2_ QDs based PDs before and after 1 months under mixed light irradiation. The inset is a partially enlarged view of the circled area.

Figure 5a displays the typical on/off behavior of SnS_2_ QDs in 0.1 M Na_2_SO_4_ at level *VI* and 0.6 V under the irradiation of a 400 nm laser. The photocurrent response as a function of wavelength is similar to the results observed in the KOH electrolyte. Based on the DFT results in Figure 3b, once SnS_2_ QDs adsorbs with the OH and SO_4_^2−^ groups, e.g. can be rapidly modulated from 1.50 (four−layer SnS_2_) to 0.552 (OH group) and 0.520 eV (SO_4_^2−^ group). The experimental results in Figure 5a demonstrated that the photo-response performance in 0.1 M Na_2_SO_4_ (3.15 μA/cm^2^) was slightly lower than that in the 0.1 M KOH (3.55 μA/cm^2^) electrolyte under a 365 nm laser at level *VI*, which is consistent with the above theoretical calculations. In addition, the resistance (*R*) at the interface between the electrolyte and electrode, Figure S4 shows the order *R*_0.1 M_ (KOH) > *R*_0.1 M_ (Na_2_SO_4_) > *R*_0.1 M_ (HCl). This implies that the type of electrolyte has a strong influence on the photoresponsive behavior, which can be attributed to the synergy between the atomic structure of SnS_2_ and the adsorbed functional groups. In addition, the *P*_ph_ in the HCl electrolyte was negligible compared to that in the 0.1 M KOH and Na_2_SO_4_ electrolytes. Therefore, we mainly investigated the photoresponse behavior of SnS_2_ QDs-based PDs in KOH and Na_2_SO_4_ electrolytes.

The response (*t*_res_) and recovery (*t*_rec_) time are other key parameters for PDs in practical applications. Regarding SnS_2_ QDs-based PDs, the *t*_res_/*t*_rec_ are measured under the irradiation of 365, 400, 475 and 550 nm at level *VI* in 0.1 M KOH and 0.6 V as demonstrated in Figure 5b. There was no much difference in *t*_res_/*t*_rec_ under different test conditions, indicating a fast photoresponse behavior of SnS_2_ QDs-based PDs under various wavelengths. The estimated *t*_res_/*t*_rec_ under the irradiation of 475 nm are 0.11/0.13 s, which are lower than most currently reported PEC-type PDs, including G_2_O_3_ films [69], BP NSs [70] and GaSe nanoflakes [71]. As shown in Figure 5c, the electrochemical impedance spectroscopy (EIS) of SnS_2_ QDs electrodes in different concentrations of KOH electrolytes revealed the recombination dynamics at the PD-electrolyte interface under the darkness. The interfacial resistance between the PDs and electrolytes decreased with the increase of KOH concentrations. Smaller interfacial resistance values facilitate electron transfer and thus improve the photovoltaic performance [72].

The long-term stability of SnS_2_ QDs-based PDs was evaluated both initially and after 1 months in 0.1 M KOH under mixed light. Figure 5d indicates no significant degradation for virgin SnS2 QDs samples (Figure 4d), which decreased by approximately 32.21% after 1 month and remained at an acceptable level. As the test time continuously increasesd, *P*_ph_ exhibited a gradual decrease, mainly caused by the detachment of the SnS_2_ QDs sample from the ITO glass substrate. The long-term stability of SnS_2_ QDs can be effectively utilized for practical PEC-type devices, including new nucleic acid tests [73], biosensors [74] and bioanalysis [75].

## 4 Conclusions

In summary, ultrasmall and high-quality SnS_2_ QDs with a uniform horizontal dimension of 3.17 nm and thickness of 2.39 nm (four layers) were successfully prepared by combining top–down and bottom–up approaches. The as-prepared SnS_2_ QDs was employed to make a series of PEC-type photodetectors (PDs), which were systematically characterized under varying monochromatic lights, electrolytes and bias voltages. The SnS_2_ QDs-based PDs exhibited a high responsivity of 0.86 mA/W, a high detectivity of 5.83 × 10^9^ Jones, and good long-term stability, which are better than most previously reported PEC-type PDs. The excellent and stable periodic optoelectronic performance of SnS_2_ QD-based PDs in different electrolytes was explained by the corresponding band structures using density functional theory. The facile approach of preparing the SnS_2_ QDs can be employed for the preparation of other low-dimensional metal dichalcogenides QDs with high optoelectronic performances.

## Supplementary Material

Supplementary Material Details

## References

[1] Xia F., Mueller T., Lin Y. M. (2009). Ultrafast graphene photodetector. Nat. Nanotechnol..

[2] Kang P., Wang M. C., Knapp P. M. (2016). Crumpled graphene photodetector with enhanced, strain-tunable, and wavelength-selective photoresponsivity. Adv. Mater..

[3] Youngblood N., Chen C., Koester S. J. (2015). Waveguide-integrated black phosphorus photodetector with high responsivity and low dark current. Nat. Photonics.

[4] Chen X., Lu X., Deng B. (2017). Widely tunable black phosphorus mid-infrared photodetector. Nat. Commun..

[5] Mak K. F., Shan J. (2016). Photonics and optoelectronics of 2D semiconductor transition metal dichalcogenides. Nat. Photonics.

[6] Wang Q. H., Kalantar-Zadeh K., Kis A. (2012). Electronics and optoelectronics of two-dimensional transition metal dichalcogenides. Nat. Nanotechnol..

[7] Hwang E. H., Das Sarma S. (2008). Acoustic phonon scattering limited carrier mobility in two-dimensional extrinsic graphene. *Phys. Rev. B*.

[8] Gusmao R., Sofer Z., Pumera M. (2017). Black phosphorus rediscovered: from bulk material to monolayers. Angew. Chem. Int. Ed. Engl..

[9] Hu C. X., Xiao Q., Ren Y. Y. (2018). Polymer ionic liquid stabilized black phosphorus for environmental robust flexible optoelectronics. *Adv. Funct. Mater.*.

[10] Duan X., Wang C., Pan A. (2015). Two-dimensional transition metal dichalcogenides as atomically thin semiconductors: opportunities and challenges. Chem. Soc. Rev..

[11] Lee C., Li Q., Kalb W. (2010). Frictional characteristics of atomically thin sheets. Science.

[12] Tongay S., Sahin H., Ko C. (2014). Monolayer behaviour in bulk ReS_2_ due to electronic and vibrational decoupling. *Nat. Commun.*.

[13] Radisavljevic B., Radenovic A., Brivio J. (2011). Single-layer MoS_2_ transistors. Nat. Nanotechnol..

[14] Lee I., Kang W. T., Kim J. E. (2020). Photoinduced tuning of Schottky barrier height in graphene/MoS_2_ heterojunction for ultrahigh performance short channel phototransistor. ACS Nano.

[15] Yao J., Zheng Z., Yang G. (2017). All-layered 2D optoelectronics: a high-performance UV−vis−NIR broadband SnSe photodetector with Bi_2_Te_3_ topological insulator electrodes. Adv. Funct. Mater..

[16] Gonzalez J. M., Oleynik I. I. (2016). Layer-dependent properties of SnS_2_ and SnSe_2_ two-dimensional materials. *Phys. Rev. B*.

[17] Fan C., Li Y., Lu F. Y. (2016). Wavelength dependent UV–Vis photodetectors from SnS_2_ flakes. RSC Adv..

[18] Hosseini S. A., Esfandiar A., Zad A. I. (2019). High-photoresponsive backward diode by two-dimensional SnS_2_/silicon heterostructure. ACS Photonics.

[19] Jia X. S., Tang C. C., Pan R. H. (2018). Thickness-dependently enhanced photodetection performance of vertically grown SnS_2_ nanoflakes with large size and high production. ACS Appl. Mater. Interfaces.

[20] Kufer D., Nikitskiy I., Lasanta T. (2015). Hybrid 2D-0D MoS2 -PbS quantum dot photodetectors. Adv. Mater..

[21] Kang D.-H., Pae S. R., Shim J. (2016). An ultrahigh-performance photodetector based on a perovskite-transition-metal-dichalcogenide hybrid structure. Adv. Mater..

[22] Ren Z., Sun J., Li H. (2017). Bilayer PbS quantum dots for high-performance photodetectors. Adv. Mater..

[23] de Arquer F. P. G., Talapin D. V., Klimov V. I. (2021). Semiconductor quantum dots: technological progress and future challenges. Science.

[24] Sriram P., Wen Y. P., Manikandan A. (2020). Enhancing quantum yield in strained MoS_2_ bilayers by morphology-controlled plasmonic nanostructures toward superior photodetectors. Chem. Mater..

[25] Kim J., Heo K., Kang D. H. (2019). Rhenium diselenide (ReSe_2_) near-infrared photodetector: performance enhancement by selective p-doping technique. *Adv. Sci.*.

[26] Li S., Chen X., Liu F. (2019). Enhanced performance of a CVD MoS_2_ photodetector by chemical in situ n-type doping. ACS Appl. Mater. Interfaces.

[27] Medintz I. L., Uyeda H. T., Goldman E. R. (2005). Quantum dot bioconjugates for imaging, labelling and sensing. Nat. Mater..

[28] Wise F. W. Lead salt quantum dots: the limit of strong quantum confinement. Acc. Chem. Res..

[29] Olkhovets A., Hsu R. C., Lipovskii A. (1998). Size-dependent temperature variation of the energy gap in lead-salt quantum dots. Phys. Rev. Lett..

[30] Yuan Y., Cheng X., Bao T. (2015). Tungsten sulfide quantum dots as multifunctional nanotheranostics for in vivo dual-modal image-guided photothermal/radiotherapy synergistic therapy. ACS Nano.

[31] Yin W., Liu X., Zhang X. (2020). Synthesis of tungsten disulfide and molybdenum disulfide quantum dots and their applications. Chem. Mater..

[32] Ding X., Peng F., Zhou J. (2019). Defect engineered bioactive transition metals dichalcogenides quantum dots. Nat. Commun..

[33] Fu X., Ilanchezhiyan P., Kumar G. M. (2017). Tunable UV−visible absorption of SnS_2_ layered quantum dots produced by liquid phase exfoliation. Nanoscale.

[34] Lei Y. M., Zhou J., Chai Y. Q. (2018). SnS_2_ quantum dots as new emitters with strong electrochemiluminescence for ultrasensitive antibody detection. Anal. Chem..

[35] Kresse G., Furthmiiller J. (1996). Efficiency of ab-initio total energy calculations for metals and semiconductors using a plane-wave basis set. Comput. Mater. Sci..

[36] Perdew J. P., Chevary J. A., Vosko S. H. (1992). Atoms, molecules, solids, and surfaces: applications of the generalized gradient approximation for exchange and correlation. Phys. Rev. B Condens. Matter.

[37] Blöchl P. E. (1994). Projector augmented-wave method. Phys. Rev. B.

[38] Monkhorst H. J., Pack J. D. (1976). Special points for Brillouin-zone integrations. Phys. Rev. B.

[39] Burton L. A., Whittles T. J., Hesp D. (2016). Electronic and optical properties of single crystal SnS_2_: an earth-abundant disulfide photocatalyst. J. Mater. Chem. A.

[40] Grimme S., Antony J., Ehrlich S. (2010). A consistent and accurate ab initio parametrization of density functional dispersion correction (DFT-D) for the 94 elements H-Pu. J. Chem. Phys..

[41] Ahn J. H., Lee M. J., Heo H. (2015). Deterministic two-dimensional polymorphism growth of hexagonal n-type SnS_2_ and orthorhombic p-type SnS crystals. Nano Lett..

[42] Voznyi A., Kosyak V., Opanasyuk A. (2016). Structural and electrical properties of SnS_2_ thin films. Mater. Chem. Phys..

[43] Xia J., Zhu D., Wang L. (2015). Large-scale growth of two-dimensional SnS_2_ crystals driven by screw dislocations and application to photodetectors. Adv. Funct. Mater..

[44] Huang Y., Sutter E., Sadowski J. T. (2014). Tin disulfide-an emerging layered metal dichalcogenide semiconductor: materials properties and device characteristics. ACS Nano.

[45] Song H. S., Li S. L., Gao L. (2013). High-performance top-gated monolayer SnS_2_ field-effect transistors and their integrated logic circuits. Nanoscale.

[46] Kang J., Wood J. D., Wells S. A. (2015). Solvent exfoliation of electronic-grade, two-dimensional black phosphorus. ACS Nano.

[47] Zhou X., Zhang Q., Gan L. (2016). Large-size growth of ultrathin SnS_2_ nanosheets and high performance for phototransistors. Adv. Funct. Mater..

[48] Di T. M., Zhu B. C., Cheng B. (2017). A direct Z-scheme g-C_3_N_4_/SnS_2_ photocatalyst with superior visible-light CO_2_ reduction performance. J. Catal..

[49] Kim M. J., Jeon S. J., Kang T. W. (2017). 2H-WS_2_ Quantum dots produced by modulating the dimension and phase of 1T-nanosheets for antibody-free optical sensing of neurotransmitters. ACS Appl. Mater. Interfaces.

[50] Ma R. Z., Sasaki T. (2015). Two-dimensional oxide and hydroxide nanosheets: controllable high-quality exfoliation, molecular assembly, and exploration of functionality. Acc. Chem. Res..

[51] Eads C. N., Bandak D., Neupane M. R. (2017). Anisotropic attosecond charge carrier dynamics and layer decoupling in quasi-2D layered SnS_2_. Nat. Commun..

[52] Fang J. X., Chen M. N., Fang Z. (2017). Thickness-dependent photoelectrochemical property of tin disulphide nanosheets. Micro Nano Lett..

[53] Lopez-Sanchez O., Lembke D., Kayci M. (2013). Ultrasensitive photodetectors based on monolayer MoS_2_. Nat. Nanotechnol..

[54] Mak K. F., Lee C., Hone J. (2010). Atomically thin MoS: a new direct-gap semiconductor. Phys. Rev. Lett..

[55] Friedrich R., Ghorbani-Asl M., Curtarolo S. (2022). Data-driven quest for two-dimensional non-van der waals materials. Nano Lett..

[56] Da Silva J. L. F., Ganduglia-Pirovano M. V., Sauer J. (2007). Hybrid functionals applied to rare-earth oxides: the example of ceria. *Phys. Rev. B*.

[57] Hinuma Y., Gruneis A., Kresse G. (2014). Band alignment of semiconductors from density-functional theory and many-body perturbation theory. *Phys. Rev. B*.

[58] Xing C., Chen X., Huang W. (2018). Two-dimensional lead monoxide: facile liquid phase exfoliation, excellent photoresponse performance, and theoretical investigation. ACS Photonics.

[59] Huang W., Jiang X., Wang Y. (2018). Two-dimensional beta-lead oxide quantum dots. Nanoscale.

[60] Gao L., Chen H., Wang R. (2021). Ultra-small 2D PbS nanoplatelets: liquid-phase exfoliation and emerging applications for photo-electrochemical photodetectors. *Small*.

[61] Huang W., Xing C., Wang Y. (2018). Facile fabrication and characterization of two-dimensional bismuth(III) sulfide nanosheets for high-performance photodetector applications under ambient conditions. Nanoscale.

[62] Huang W., Xie Z., Fan T. (2018). Black-phosphorus-analogue tin monosulfide: an emerging optoelectronic two-dimensional material for high-performance photodetection with improved stability under ambient/harsh conditions. J. Mater. Chem. C.

[63] Fan X., Su L., Zhang F. (2019). Layer-dependent properties of ultrathin GeS nanosheets and application in UV–Vis photodetectors. ACS Appl. Mater. Interfaces.

[64] Yang X., Qu L., Gao F. (2022). High-performance broadband photoelectrochemical photodetectors based on ultrathin Bi_2_O_2_S nanosheets. ACS Appl. Mater. Interfaces.

[65] Li Z., Qiao H., Guo Z. (2018). High-performance photo-electrochemical photodetector based on liquid-exfoliated few-layered InSe nanosheets with enhanced stability. Adv. Funct. Mater..

[66] Ma D., Zhao J., Wang R. (2019). Ultrathin GeSe nanosheets: from systematic synthesis to studies of carrier dynamics and applications for a high-performance UV–Vis photodetector. ACS Appl. Mater. Interfaces.

[67] Gao L., Wang R., Kuklin A. V. (2021). PbSe nanocrystals produced by facile liquid phase exfoliation for efficient UV–Vis photodetectors. Adv. Funct. Mater..

[68] Zhang Y., You Q., Huang W. (2020). Few-layer hexagonal bismuth telluride (Bi_2_Te_3_) nanoplates with high-performance UV–Vis photodetection. Nanoscale Adv..

[69] Huang L., Hu Z., Zhang H. (2021). A simple, repeatable and highly stable self-powered solar-blind photoelectrochemical-type photodetector using amorphous Ga_2_O_3_ films grown on 3D carbon fiber paper. J. Mater. Chem. C.

[70] Ren X., Li Z., Huang Z. (2017). Environmentally robust black phosphorus nanosheets in solution: application for self-powered photodetector. Adv. Funct. Mater..

[71] Bianca G., Zappia M. I., Bellani S. (2020). Liquid-phase exfoliated GeSe nanoflakes for photoelectrochemical-type photodetectors and photoelectrochemical water splitting. ACS Appl. Mater. Interfaces.

[72] Zhang Y., Huang P., Guo J. (2020). Graphdiyne-based flexible photodetectors with high responsivity and detectivity. Adv. Mater..

[73] Zeng R., Zhang L., Su L. (2019). Photoelectrochemical bioanalysis of antibiotics on rGO-Bi_2_WO_6_-Au based on branched hybridization chain reaction. Biosens. Bioelectron..

[74] Wang M., Yin H., Zhou Y. (2019). Photoelectrochemical biosensor for microRNA detection based on a MoS_2_/g-C_3_N_4_/black TiO_2_ heterojunction with Histostar@AuNPs for signal amplification. Biosens. Bioelectron..

[75] Gill R., Zayats M., Willner I. (2008). Semiconductor quantum dots for bioanalysis. Angew. Chem., Int. Ed. Engl..

