# Supplementary material for: Ultrasmall SnS2 quantum dot−based photodetectors with high responsivity and detectivity
Source: Nanophotonics. 2022 Sep 29;11(21):4781–92. doi: 10.1515/nanoph-2022-0277 (PMC11502062; doi:10.1515/nanoph-2022-0277)
Supplement: Supplementary file 1 — Supplementary Material Details [file j_nanoph-2022-0277_suppl_001.docx]

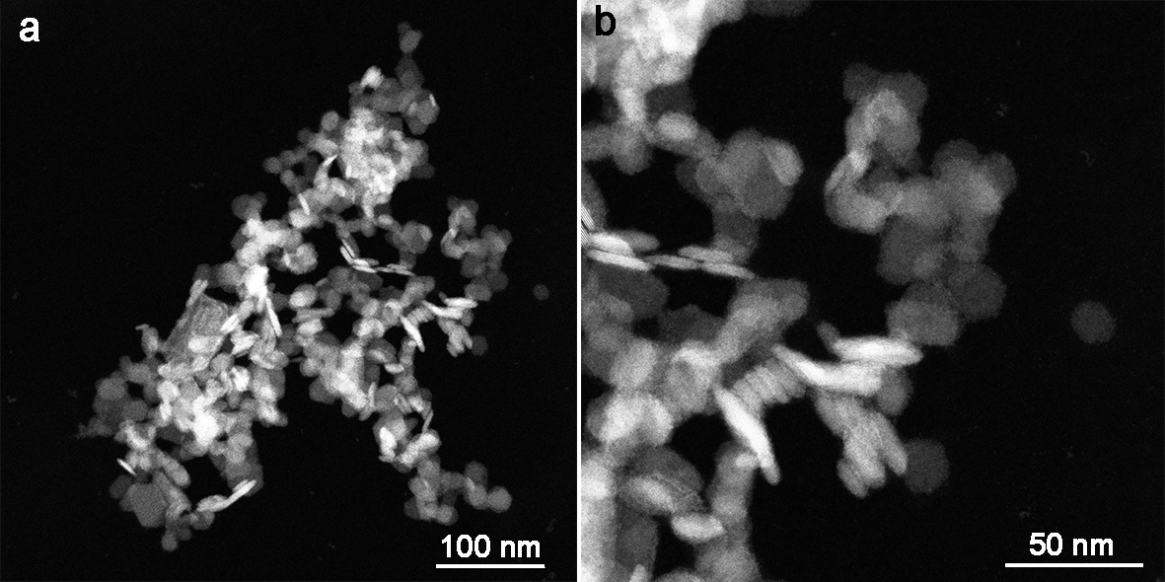
**Figure S1** a, b) TEM images of SnS_2_ nanosheets.


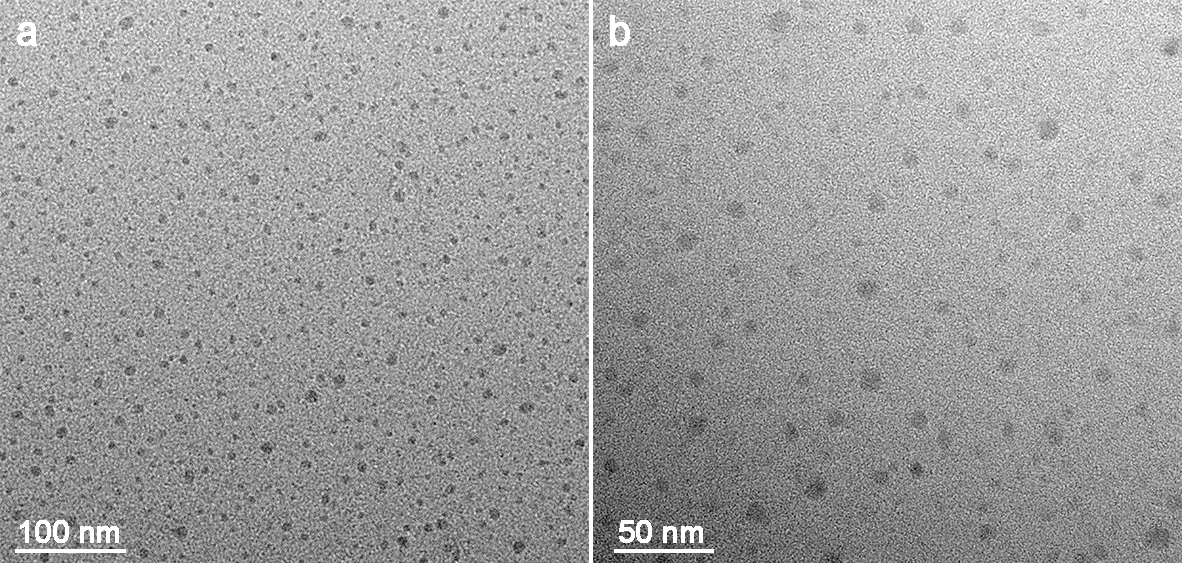
**Figure S2**. a, b) TEM images of SnS_2_ QDs after 1 month.


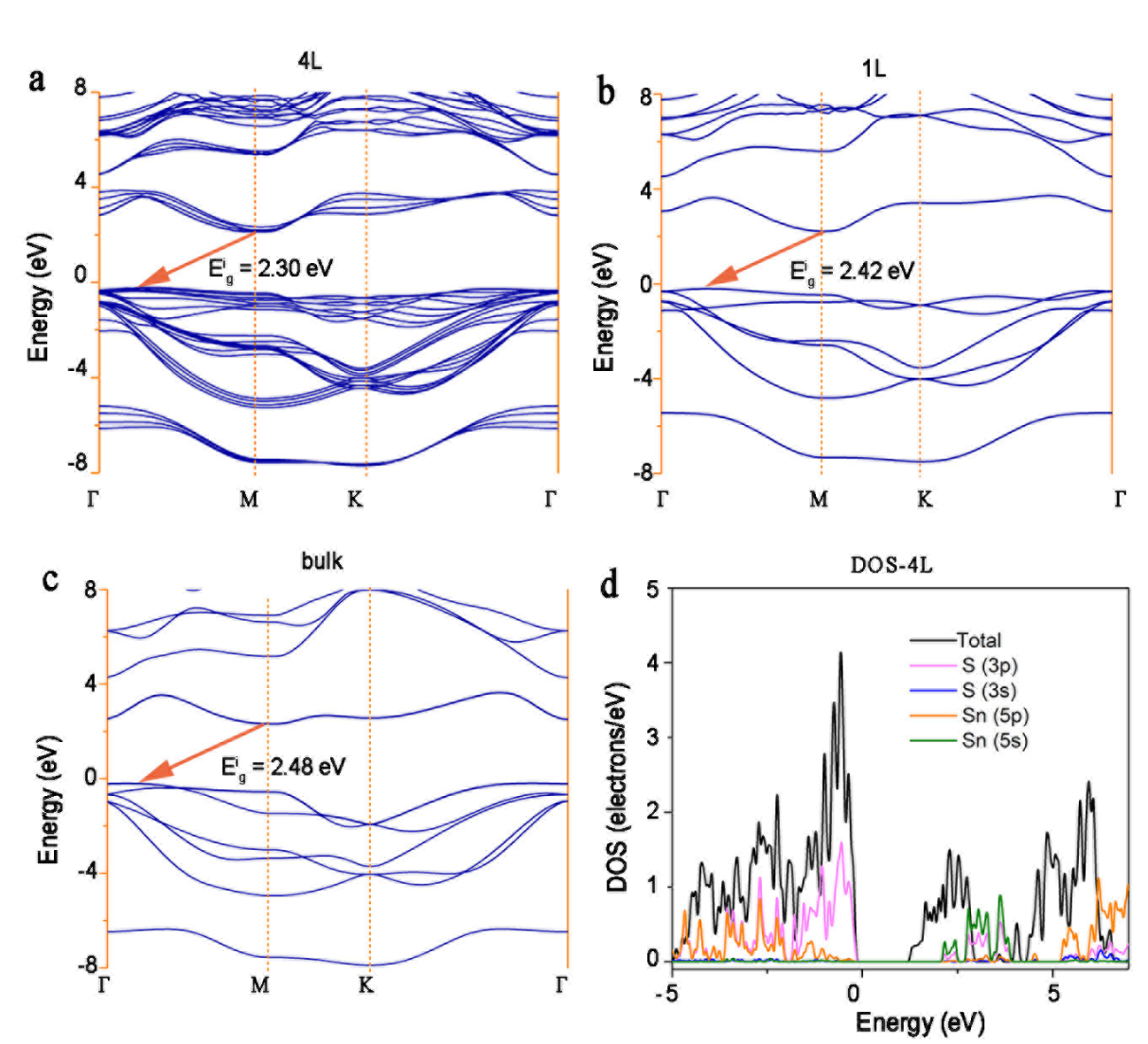


**Figure S3.** The band structures of SnS_2_ with layer number of a) 4L, b) 1L, c) bulk calculated by HSE hybrid functional. d) The electronic density of states (DOS) for 4L SnS_2_.


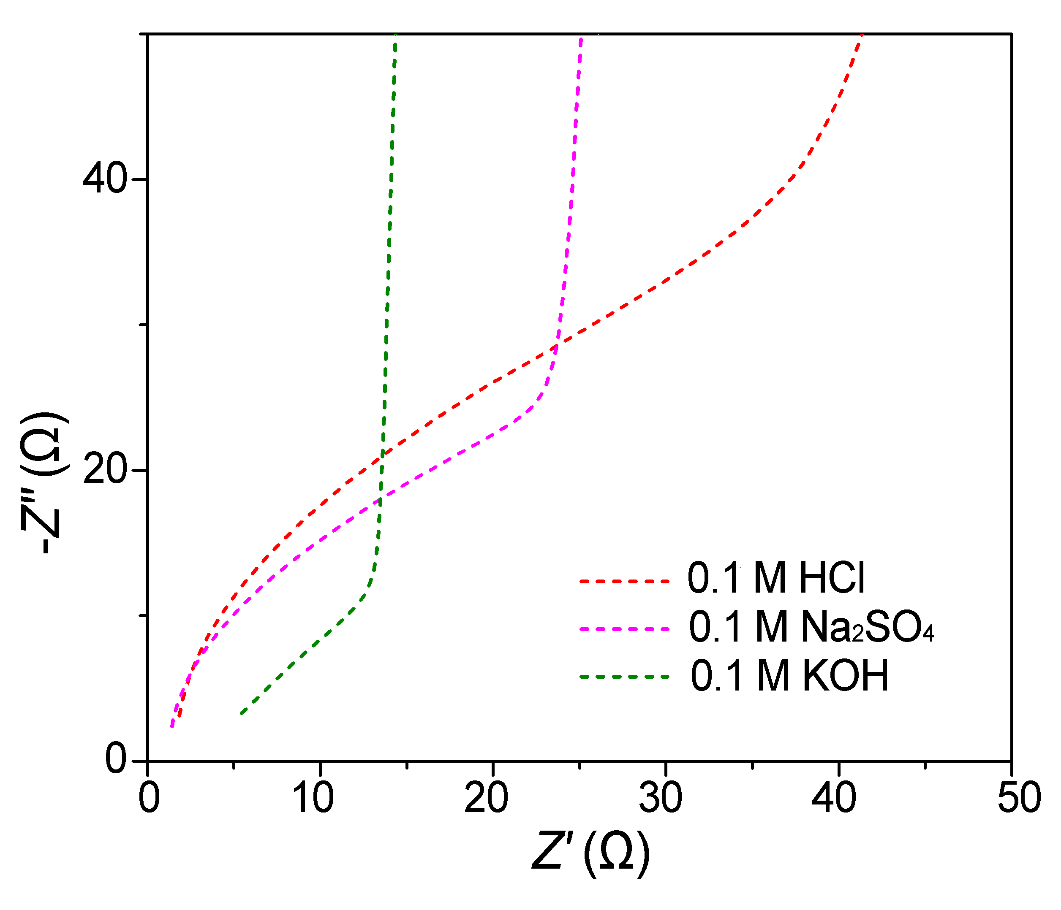


**Figure S4.** EIS results of SnS_2_ QDs-based PDs in different electrolytes under the darkness.

**Table S1.** The calculated band gaps of bulk and few-layer SnS2 by different functionals.

| Layer (L) Functional a lattice parameter (Å) Bandgap (eV) |  |
| --- | --- |

1 PBE/HSE 3.697 1.588/2.420

2 PBE 3.662 1.528

4 PBE/HSE 3.660 1.504/2.300

8 PBE 3.667 1.277

12 PBE 3.663 1.324

Bulk PBE/HSE 3.667 1.629/2.480

4L-OH PBE 3.660 0.552

4L-2OH PBE 3.660 0.520

4L-SO_4_  PBE 3.660 0.701

4L-H PBE 3.660 0.630

|  |
| --- |

**Table S2.** The light power density (*P*_λ_) of incident light with various wavelengths and mixed light.

| *P*_λ_ (mW/cm^2^) mixed light 365 nm 380 nm 400 nm 475 nm 550 nm |
| --- |
| Ⅰ 32.00 0.073 0.073 0.90 1.67 1.76  Ⅱ 56.80 0.40 1.15 2.33 3.68 3.71  Ⅲ 193.00 4.85 6.97 10.35 13.93 13.30  Ⅳ 347.00 10.25 13.38 19.26 25.60 24.00  Ⅴ 449.10 14.00 17.83 25.30 33.60 32.00  Ⅵ 585.00 19.15 23.80 33.20 44.10 41.71 |

**Table S3.** The measured photocurrent density (*P*ph) of SnS_2_ QDs-based PDs at various wavelength in 0.1 and 0.5 M KOH.

| *P*_ph_−0.1 M (μA cm^-2^) 365 nm 380 nm 400 nm 475 nm 550 nm |
| --- |
| Ⅰ 0.75 0.25 0.12 0.16 0.04  Ⅱ 1.58 0.72 0.37 0.31 0.06  Ⅲ 2.30 1.20 0.56 0.46 0.08  Ⅳ 3.08 1.52 0.73 0.60 0.09  Ⅴ 3.25 1.58 0.75 0.62 0.10  Ⅵ 3.55 1.85 0.83 0.64 0.11 |

*P*_ph_−0.5 M (μA cm^-2^) 365 nm 380 nm 400 nm 475 nm 550 nm

| Ⅰ 1.50 0.88 0.62 0.44 0.08  Ⅱ 3.70 1.42 0.83 0.68 0.12  Ⅲ 8.82 3.09 2.34 1.81 0.31  Ⅳ 13.81 5.50 3.08 2.58 0.52  Ⅴ 14.83 6.25 3.51 3.04 0.61  Ⅵ 16.38 7.41 3.90 3.36 0.72 |
| --- |
|  |
